# Identifying Social Learning in Animal Populations: A New ‘Option-Bias’ Method

**DOI:** 10.1371/journal.pone.0006541

**Published:** 2009-08-06

**Authors:** Rachel L. Kendal, Jeremy R. Kendal, Will Hoppitt, Kevin N. Laland

**Affiliations:** 1 Centre for Social Learning and Cognitive Evolution, School of Psychology, University of St Andrews, St Andrews, Fife, United Kingdom; 2 Centre for Social Learning and Cognitive Evolution, School of Biology, University of St Andrews, St Andrews, Fife, United Kingdom; Lund University, Sweden

## Abstract

**Background:**

Studies of natural animal populations reveal widespread evidence for the diffusion of novel behaviour patterns, and for intra- and inter-population variation in behaviour. However, claims that these are manifestations of animal ‘culture’ remain controversial because alternative explanations to social learning remain difficult to refute. This inability to identify social learning in social settings has also contributed to the failure to test evolutionary hypotheses concerning the social learning strategies that animals deploy.

**Methodology/Principal Findings:**

We present a solution to this problem, in the form of a new means of identifying social learning in animal populations. The method is based on the well-established premise of social learning research, that - when ecological and genetic differences are accounted for - social learning will generate greater homogeneity in behaviour between animals than expected in its absence. Our procedure compares the observed level of homogeneity to a sampling distribution generated utilizing randomization and other procedures, allowing claims of social learning to be evaluated according to consensual standards. We illustrate the method on data from groups of monkeys provided with novel two-option extractive foraging tasks, demonstrating that social learning can indeed be distinguished from unlearned processes and asocial learning, and revealing that the monkeys only employed social learning for the more difficult tasks. The method is further validated against published datasets and through simulation, and exhibits higher statistical power than conventional inferential statistics.

**Conclusions/Significance:**

The method is potentially a significant technological development, which could prove of considerable value in assessing the validity of claims for culturally transmitted behaviour in animal groups. It will also be of value in enabling investigation of the social learning strategies deployed in captive and natural animal populations.

## Introduction

Social learning, or learning from others, is of widespread current interest because it potentially provides a means by which animals can acquire adaptive information about their environment rapidly and efficiently. Social learning is thought to underlie the rapid diffusion of novel behavioural variants, inter-population variation in behaviour, and cultural traditions, in animals from fishes to apes [1]–[4]. Here, we use the term ‘culture’ in its broadest sense to refer to any instance of social transmission of behaviour, regardless of the underlying social learning process [5], [but see 6 for alternative definitions of ‘culture’].

Interest in animal social learning has also been fuelled by reports of intra- and inter-population variation in the behavioural repertoires of animal populations, spawning claims of ‘culture’ in apes [7], [3], [8] cetaceans [4], [9], and monkeys [10]. However, claims that this data demonstrate animal cultures remain controversial in the absence of clear methods for ruling out alternative explanations for the variation, such as genetic differences between populations, or asocial/individual learning in response to differing environmental conditions [11], [5]. Moreover, as learning is frequently functional, adaptive, based on genetic proclivities, and responsive to ecological resources, the current ‘ethnographic’ method, which proclaims culture where the alternatives can be dismissed, is vulnerable to excluding genuine cases of social learning. Thus researchers currently lack tools for identifying social learning in a naturalistic context (i.e. in animal groups, whether wild or captive). In this paper we introduce a new method to add to the researcher’s toolbox for identifying culture in the wild, the main benefit of which is that it provides an estimate of the probability that a putative tradition can be explained in the absence of social learning. This allows individual cases to be accepted or rejected as socially transmitted according to field-wide consensual standards for probability estimates (i.e. *α*<0.05) rather than subjective judgements of plausibility reliant on opinions that vary widely amongst practitioners.

Relatively few experimental attempts have been made to examine social learning processes in social settings. The use of translocation experiments, such as the transfer of individuals between populations, effective for fishes [2], is often not logistically possible or ethically acceptable for other taxa, especially primates. Experiments based on demonstrator-observer pairings [12], transmission chains [13] and seeded diffusions [14], while effective in captivity, are typically unable to isolate social learning in natural populations. Attempts to use statistical analysis of the shape of the diffusion curve to infer the presence of social and asocial learning have been discredited [15], [16]. While other methods [e.g. phylogenetic approaches], [ 17; network–based diffusion analysis, 18] exhibit promise, none are yet well-established and validated in this domain, and there is wide recognition of the need for new methods [5], [6].

This methodological dearth has proven a further impediment to research on animal social learning, since it has hindered the testing of evolutionary hypotheses concerning the circumstances under which individuals should exploit the cheap (relative to asocial learning) but potentially unreliable information acquired through social learning. Game theory and population genetic models predict that natural selection ought to have fashioned specific adaptive social learning strategies that dictate the contexts under which individuals will exploit information provided at a cost by others [19], [20], [21] in order to circumvent the risk of acquiring unreliable information. Plausible strategies include copying when asocial learning would be costly, copying in proportion to the demonstrator’s payoff, or copying when dissatisfied with the current payoff. Although learning strategies have been investigated in captive animals [22], [23], hitherto it has proven difficult to do so in a natural context without a means of isolating social learning.

Here we describe a novel method that allows social and asocial learning to be distinguished inferentially, in animal datasets that record the spread of novel behaviour patterns. The method is applicable to natural, semi-natural and captive social groups of animals, and examines the relative frequency of learned behavioural variants (or ‘options’), performed in a particular ecological or social context. The analysis rests on the commonly applied premise of social learning research, the assumption that – when ecological and genetic differences are accounted for - social learning will generate a greater within-population homogeneity in the option choices exhibited for a given diffusion (henceforth an ‘option bias’) than expected in the absence of social learning.

For example, when probing for termites in their mound, chimpanzees are reported to use either a short- or long-twig method [3]. If this behaviour is learned socially then a given population may disproportionately use one method, whereas if it is learned asocially one might expect use of both methods in proportion to their opportunity and profitabilities. Thus, provided alternative forms of bias can be ruled out (see discussion), the level of homogeneity of behaviour within a population potentially provides a metric that can be used probabilistically to detect a social influence on learning. In order to test for social learning in the observed data, however, the probability that option biases of the magnitude observed in the actual data could be the result of chance or asocial learning alone must be computed. Here we present a comparison of the observed option bias against a sampling distribution bootstrapped by randomizing the observed data. (We also consider a Monte Carlo simulation approach but our analysis finds randomization is typically more powerful. See Supplementary Material (S1) for details). We are able to reject the null hypothesis, that the observed option bias is the result of stochastic or asocial learning processes, if the magnitude of the option bias calculated from the observed data exceeds that which could be reasonably expected through chance and asocial learning alone (that is, if it lies within the upper 5% tail of the bootstrapped distribution reliant solely on asocial processes). The option bias methodology can therefore be thought of as a rigorous, quantitative version of the ethnographic method, by providing a probability that the observed levels of within-group homogeneity arose in the absence of social learning, and thereby allows judgements as to the plausibility of a social learning explanation to be based on consensual standards, rather than subjective judgements. The method is designed to be sympathetic to the constraints of data collection from natural populations, and can be used, in particular, when standard inferential statistics are inappropriate because of low power and non-independence within the data. Critically, the analysis does not require large population sample sizes, large numbers of populations or complete datasets to be effective, nor knowledge of the likelihood of each option's use in the absence of social learning. Likewise the phase of social transmission relative to the innovation event [24] need not be known. Like inferential statistical tests, and statistical approaches in general, the method is appropriate for specific forms of data, and may generate misleading conclusions if applied outside of this domain. The method requires the researcher to independently (*i*) assess the role of genetic differences between populations, (*ii*) assess any population differences in ecology or option profitability's that may effect the use of behavioural options through asocial learning, (*iii*) identify the behavioural variants or ‘options’ to perform the task in question, and (*iv*) identify the populations for which homogeneity of behaviour is expected (see discussion). While the method is not invalidated by genetic or ecological heterogeneity across populations, in such instances it either requires independent estimates of the probability of each option in each population, which in some instances may be difficult to obtain, or application on a smaller scale, within which such sources of bias do not apply. The analysis is also inappropriate in those rare instances where social learning is not expected to generate behavioural homogeneity.

Here, we apply the method to data collected from callitrichid monkeys, in order to evaluate a hypothesis [19], [25] concerning the circumstances under which animals, including humans, rely on social learning as opposed to their own direct (asocial) experience. On the basis of a theoretical analysis, Boyd & Richerson [25] predicted that animals increasingly rely on social information as the costs of asocial learning (e.g. temporal, energetic) increase. Thus, animals should rely on asocial learning if these costs are low due to the potential unreliability of information gained through social learning. Our analysis of the spread of novel foraging behaviour in 26 small groups of callitrichid monkeys concludes that some but not all novel solutions are socially transmitted. Furthermore, we confirm that the solutions to complex tasks are indeed more likely to spread through social learning than those for simple tasks. We also compare the power of different statistical analyses applied to simulated option-bias data and conduct a validation exercise, using data from two conventional social learning experiments, which supports the reliability of our method. Finally, we provide the necessary computer script in R for others to implement the method and adapt it for their own individual circumstances (see Supplementary Material, S2).

## Methods

### Ethics Statement

It was not necessary to purchase any animals for the project, since we used established zoo (Jersey, Twycross, Banham, Whipsnade, Marwell) populations. The callitrichid monkeys were all captive bred, either directly at the focal zoo or at other zoos. The zoos took full responsibility for the import, breeding, housing, general husbandry, feeding and the health of the animals. As the project involved only behavioural data collection, no U.K. Home Office licensed procedures, transgenic procedures or cloning were involved in the experimentation and the animals were not subjected to any pain, stress or direct handling. To the contrary, the experimentation, which merely involved introducing novel foraging tasks into the regular zoo enclosures, was regarded by the zoos as providing enrichment to the animals, and such procedures are generally widely associated with increased health and happiness in zoo animals. Hence, the monkeys were not exposed to any suffering as a result of the project, and the experimentation is thought to have improved their welfare. All experimental work was conducted in the full knowledge and collaboration of zoo staff, was approved by their ethics boards and met *Association for the Study of Animal Behaviour* (ASAB) and U.K. Home Office (HO) ethical guidelines for animal experimentation, and was in accordance with the Amsterdam protocol on animal protection and welfare. In addition, the procedures were developed in liaison with the School of Zoology's Ethics Officer at the University of Cambridge, U.K.

### Experimental Subjects and Apparatus

We studied twenty-six monospecific groups of zoo-housed callitrichids (*Leontopithecus chrysomelas*, *L. rosalia*, *L. chrysopygus*, *Callithrix argentata*, *C. geoffroyi*, *Saguinus imperator*, *S. oedipus*), totalling 108 individuals across four zoos. The subjects (ranging from 6.5 months to 18.5 years) were in group sizes (2–8 individuals) and compositions (mated pairs or families) within the bounds of those seen in the wild. They were housed in a variety of different types of enclosure with a range of husbandry regimes [see 26 for full details].

**Figure 1:**
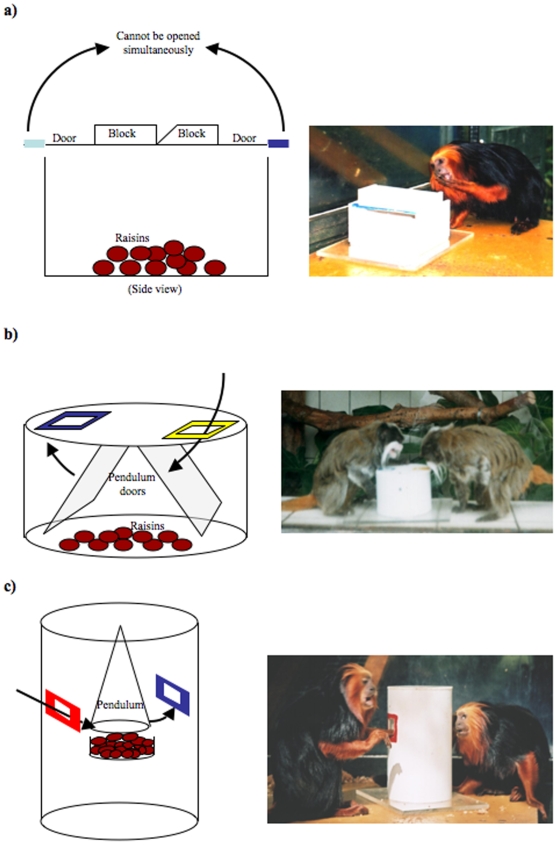
Diagrams and photos of (a) the flip-top task used by a golden headed lion tamarin, (b) the round-box task used by emperor tamarins, and (c) the cylinder task used by golden headed lion tamarins. Arrows indicate the movement of the devices that prevent simultaneous use of the task options.

Each group was exposed to three different extractive foraging tasks (each a puzzle box containing a desired food) over separate trials (i.e. one task per group per trial) in a randomised order (see Fig. 1). Pilot studies established that the tasks, labelled ‘round-box’, ‘flip-top’ and ‘cylinder’, were of increasing difficulty for the monkeys to solve. All three were opaque white plastic boxes, of varying shapes, containing raisins [see 27 for more information]. When the boxes were closed, subjects had limited visual and olfactory access to the food and had two spatially separated doors, or options, from which to extract the raisins. The options, which could not be used simultaneously, were distinguished by colour combinations (blue versus either green, red or yellow) visible to both di- and trichromatic individuals, and the order of task presentation to each group was pseudo-randomised. There was no effect of colour preference across all groups for any task (*Mann-Whitney*: flip-top: *U*_94,95_ = 4303, *p* = 0.534; cylinder: *U*_94,95_ = 4379, *p* = 0.681; round-box: *U*_94,95_ = 4403, *p* = 0.844) and no instances of scrounging were observed. Although the large size of the tasks relative to the monkeys should have eliminated any effects of handedness, any side-biases were controlled for as the orientation of the tasks was randomised across trials. The tasks were designed to be solved using foraging actions, natural to all genera, such as employed when turning over bark, exploring crevices and rummaging in leaf litter.

### Behavioural Data and Statistical Analysis

Each trial began with the presentation of a novel task to a group and lasted for 30 minutes or until all of the raisins had been extracted from the task, whichever occurred sooner. For each individual, we recorded (1) the latency to first task contact, (2) the latency to, and (3) frequency of, all unsuccessful and successful task manipulations (or food extractions), and (4) the task options used (e.g. blue or yellow) (see table 1 for definitions). Manipulations were divided into successful and unsuccessful as, with associative learning, there are reasons to expect an option bias to be stronger following reinforcement (e.g. Thorndike's ‘Law of Effect’). Here we outline our methodology using successful task manipulations as an example, but the same analysis was performed for unsuccessful manipulations. Previous analyses [26] of these data indicate that *Leontopithecus* and *Callithrix* species do not differ in the overall propensity to learn socially. Accordingly, we conduct the below option-bias analyses across genera and species.

**Table 1:** Definitions of terms as used in the context of this study.

Term	Definition
Contact	First time an individual touches the foraging task with hand or mouth.
Unsuccessful Manipulation	An individual moves part of the task but does not eat.
Successful Manipulation	An individual extracts a raisin from the task.
Learning time	Difference in latency between an individual's first ‘contact’ and first ‘successful manipulation’§.

§ Successful manipulation is assumed to be a manifestation of learning, although it is recognised that learning, in terms of retention of information, was not tested.

Option biases were analysed across 29 trials (4, 14 and 11 trials for the cylinder, flip-top and round-box, respectively), and for a total of 78 individuals. We included trials for which successful manipulations were observed, but excluding all groups made up of pairs and all trials where only one individual solved the task, since these groups do not contribute any information about option bias. All data were checked for normality using Kolmogorov-Smirnov tests and for homogeneity of variance using Levene's test. Where it was not possible to use parametric statistics, non-parametric tests were used (all two-tailed). Where multiple tests were conducted, the family-wise error rate was controlled using the Bonferroni method.

We calculated a χ^2^ value as a measure of the observed option bias for each task (henceforth the “option bias statistic”), from a contingency table of the number of manipulations for each option per group, using a null expectation of an equal number of manipulations for the two options calculated for each group (see Supplementary Material, Table S1). These data are likely to be non-independent, since most individuals made more than one manipulation within a trial, so it is not appropriate to test for social learning using a χ^2^ distribution. Instead we generate a null distribution for the option bias statistic using a randomisation approach [28]. For each randomisation, we randomly allocated individuals to groups, constraining group sizes to remain the same as that observed, and calculated the resulting option bias statistic. We repeated this procedure 10,000 times to generate a null distribution, and calculated the p-value as the proportion of the null distribution that was greater than or equal to the observed option bias statistic. Since the data are randomised at the level of individuals, the test allows for the fact that manipulations by the same individual might not be independent. In other words the observed data and the null distribution both maintain the same structure, with respect to the number of manipulations per individual, thus circumventing the problem of pseudoreplication incurred by using a χ^2^ distribution. (The Supplementary Material, S1.1, presents an alternative Monte Carlo simulation approach).

### Comparison of statistical techniques for analysing option bias

**Figure 2:**
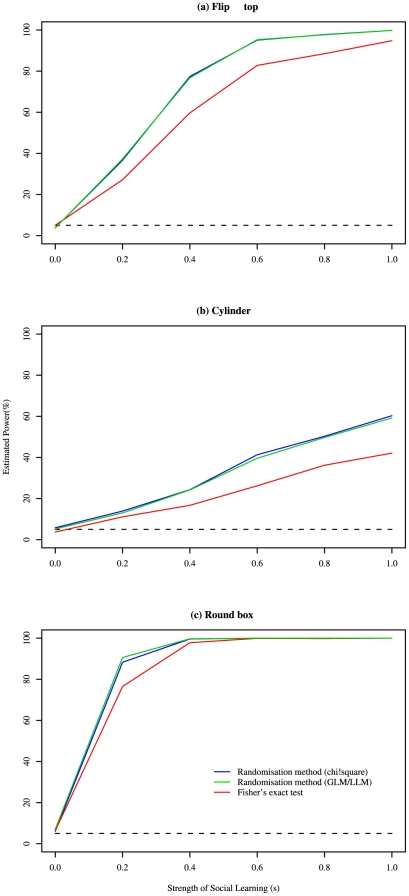
A comparison of power for three randomisation techniques (GLM and LLM represented by a single line) and Fisher's exact test. For details see text.

**Figure 3:**
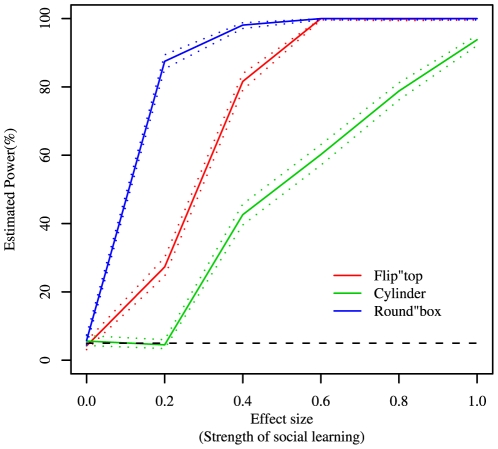
Power estimates for the option-bias method, using the χ^2^ metric, plotted against effect size for the three tasks. Dotted lines show Wilson's confidence intervals. In all cases the power is higher than that for Fisher's exact test. See supplementary material (S1.3) for details.

Randomisation is an established technique for testing hypotheses when the assumptions of conventional statistical techniques are not met [28]. Nonetheless, we wanted to validate the method in the context of detecting social learning in groups of animals. We did this using simulations of the asocial and social learning process for each task, enabling us to assess type 1 error rate and statistical power, and compare the performance of the method to other candidate techniques. These were a) Fisher's Exact Test, run on data reduced to a single datum for each individual, representing its mean response; b) a GLMM with a binomial error structure, testing for a fixed effect of group using a likeihood ratio test (LRT) with individual as a random effect; c) randomisation techniques using a test statistic generated from i) a GLM with a binomial error structure and ii) a log linear model, both with group as an explanatory variable; and d) generation of a null distribution through a Monte Carlo simulation of the asocial learning process. The simulations also allowed us to estimate the power of the chosen technique to detect a given effect size for each of the tasks (see Figures 2 and 3). Full details of the simulations can be found in the Supplementary Material (S1).

### Validating the option bias method through application to published findings

Traditional laboratory studies infer social learning by showing that an observer is more likely to solve a task using the same option as its demonstrator used compared with an alternative. If the option bias method is a suitable technique for detecting social learning in groups of animals, we would expect its findings to match those of the traditional approach when applied to the same data. Studies by Coolen et al. [29] and Whiten et al. [30] provide ideal datasets with which to validate the method. In Coolen et al.'s study (experiment 2) the authors report that, following observation of demonstrator conspecifics feeding at two food patches that differ in their profitabilities, 9-spined sticklebacks acquired a preference for the richer quality patch, whilst 3-spined sticklebacks did not. Therefore, we would expect to find a significant option bias for 9-spined sticklebacks, but not for 3-spined sticklebacks. Coolen et al. recorded the position of each fish every six seconds for ten minutes after the trial started (though here we only analyse the first 90 seconds, since the fish were tested in extinction, and we would expect an initial preference to fade over time). We reanalysed their data by treating as a successful “manipulation” any instance in which a fish was present in a food patch (or “goal zone”), and with the left or right patches being the alternative options. The fish were then assigned to groups on the basis of the demonstration they had observed: a rich right patch or a rich left patch.

A more naturalistic experimental approach to inferring social learning in groups of animals is to introduce demonstrator animals, trained to solve a task using one of two or more options, into groups of naïve individuals. Social learning is then inferred if the individuals in each group tend to adopt the method of their demonstrator. Whiten et al. [30] found that two groups of chimpanzees, when learning to gain food from the experimental apparatus, tended to use the same action, “poke” or “lift”, used by their demonstrator. We also applied the option bias method to these data, using up to the first 30 successes by each individual in the initial test phase, excluding the demonstrators [Fig 2a & b [in 29]].

## Results

### Task Difficulty

An analysis of the callitrichid data confirmed the existence of significant differences in the difficulty of the three tasks, as gauged by both latency to solve and number of unsuccessful task manipulations and successful extractions by the monkeys. Individuals produced significantly fewer successful manipulations (ANOVA: *F*_2,318_ = 14.77, *p*<0.001) with the cylinder than the flip-top (Tukey: *p*<0.001) and round-box (*p*<0.001) tasks. Accordingly, innovators (defined here as the first individual per group to solve the task) produced significantly fewer unsuccessful than successful manipulations with the round-box task (Wilcoxon: *T*_9_ = −6, *p* = 0.05). In contrast for the cylinder, where sample size was small, the greater incidence of unsuccessful versus successful manipulations approached significance (Wilcoxon: *T*_4_ = −0, *p* = <0.15), while there was no significant difference between unsuccessful and successful manipulations for the flip-top task (Wilcoxon: *T*_8_ = −13.5, NS). The latency between first contact and first success (‘learning time’, see Table 1) also differed between tasks (ANOVA: *F*_2,152_ = 4.305, *p* = 0.015), being shorter for round-box than flip-top (Tukey: *p* = 0.030) or cylinder (*p* = 0.057) tasks. The measure of learning time controls for time to first contact, and so these differences cannot be attributed to variation in task salience or neophobia to the task. These findings indicate that the cylinder was the most difficult, and the round-box the simplest, task to solve.

### Option Bias Analysis

Our option bias analysis applied to successful manipulations of the callitrichid tasks revealed evidence for social learning of the flip-top task (*Option bias*, p = 0.049), but not the cylinder task (*Option bias*, p = 0.15) nor the round-box task (*Option bias*, p = 0.84). The estimated power of these results is shown in Figure 3 and indicates that there is insufficient power to determine whether or not there is evidence for social learning with the cylinder task. No evidence for social learning was manifest in the unsuccessful manipulations, for any task (Flip-top: *Option bias*, p = 0.31; Cylinder: *Option bias*; p = 0.62; Round-box: *Option bias*, p = 0.673). Of the innovators, approximately equal numbers first used each task option (flip-top: 6 blue vs. 8 green; cylinder: 2 blue vs. 2 red; round-box: 6 blue vs. 5 yellow).

### Comparison of statistical techniques

All option bias methods had an appropriate type 1 error rate (∼5%), with the exception of those using GLMM, and those reliant on Monte Carlo simulation where *α* was unknown. For all tasks the GLMM had an inflated type 1 error rate, which we attribute to the asymptotic nature of the LRT (e.g. Flip-top: 20% estimated Type 1 error rate for 5% significance, 95% confidence interval: 17.6% to 22.6%). We therefore consider this an inappropriate method for analysing option bias. The Monte Carlo simulation had an appropriate type 1 error rate when the model learning parameter (*α*) was known (see Supplementary Material, S1.1), but this was inflated, in the more realistic case, when ***α*** was estimated from the data (e.g. Round-Box: 24% estimated Type 1 error rate for 5% significance, 95% confidence interval: 21.5% to 26.7%). The estimated power for the Monte Carlo simulation was always lower than that of the randomisation methods.

All randomisation methods had better power than Fisher's exact test, primarily because they take into account the strength of individual-level option bias, rather than just its direction. The relative performance of these tests is shown in Fig. 2. The performance of the randomisation techniques was very similar, consequently we consider any of these approaches appropriate for analysing option bias data. We chose to use the method described above (using a ***χ^2^*** metric) on the grounds that it is the simplest and requires less computation time for running power analyses. The estimated power for this method is shown in Fig. 3.

### Validation

In agreement with Coolen et al.'s [29] published findings, we found a significant option bias for 9-spined sticklebacks (*Option bias*, p = 0.026), but no significant option bias for 3-spined sticklebacks (*Option bias*, p = 0.38). Likewise, application of the option bias technique to Whiten et al.'s [30] chimpanzee data found strong evidence of an option bias (*Option bias*, p<0.001). That the option bias is able to detect social learning in this more naturalistic context supports our claim that it will be able to detect social learning of naturally occurring behavioural variants.

## Discussion

We have introduced a new method, called the *option-bias method*, for detecting social learning in animal populations. The method is based on a widely applied premise in social learning research, the assumption that - when ecological and genetic differences are accounted for - social learning will generate greater homogeneity in behaviour within groups than expected in its absence. The term ‘option bias’ refers to a greater than expected homogeneity in the learned option choices of animals, and we suggest that this signature is potentially a reliable indicator of social learning under well-specified circumstances. Our analyses reveal that the approach gives greater power to detect social learning than conventional inferential statistics, while its validity is confirmed through application to established experimental datasets.

We have applied the method to experimental data collected from groups of callitrichid monkeys provided with three novel foraging tasks. We conclude that there is compelling evidence for social learning in only one of these tasks, namely the flip-top task. It is highly unlikely that the observed option bias for successful manipulations in this task arose by chance under asocial (individual) learning alone. Conversely, given the relatively high power estimated for the round-box task (Figure 3), it seems likely that there is no social learning, or its effects are very weak for this task. Since the round-box was the easiest task, this finding is consistent with the hypothesis that social learning will only be used for difficult tasks [19]. Whilst the cylinder was found to be the most difficult task, the estimated power for this task was much lower over a plausible range of values for the strength of social learning (Figure 3). Here the low power reflects the small number of populations for which data is available, and small number of successful manipulations, rather than any intrinsic feature of the option-bias method. Therefore, we cannot rule out a meaningful role for social learning for the cylinder task, and think it likely that further data collection would demonstrate this.

These results are broadly consistent with the predictions by Boyd and Richerson [19], [25] and other researchers [e.g. 31] that animals' reliance on social information covaries with the cost (e.g. lost time and energy) of asocial learning. Here the best evidence for social learning is found in the flip-top task, while we cannot rule out social learning in the case of the cylinder; these are the two most difficult tasks. Conversely, the solution to the round box task, demonstrated to be easiest, is almost certainly acquired asocially. Plausibly, the monkeys may have adopted social learning to avoid the cost of excess expenditure of time or energy and reduced foraging success associated with asocial learning of complex tasks. The relatively high proportion of unsuccessful manipulations produced by innovators tackling the cylinder compared with those tackling the round-box implies that more trial-and-error-learning (or discovery) is involved in achieving success with the cylinder than round-box task. Where trial-and-error (asocial learning) is minimal (i.e. cheap), it is expected that individuals will trade this off against the costs (in the form of potentially unreliable information) associated with social learning [22], [23]. This interpretation is supported by a suite of recent experiments involving two-action tasks with chimpanzees that point to a positive relationship between reliance on social learning and task difficulty [31].

The observed significant bias for use of one option over the other with the flip-top task implies that the actions of demonstrating individuals drew the attention of conspecifics to a specific option, causing them to direct the majority of their manipulations towards it. The converse finding, that social learning is *not* involved in generating option biases in unsuccessful manipulations of the tasks, is consistent with the idea that animals may distinguish between functionally relevant and irrelevant information, or rewarded and unrewarded information, and preferentially learn about the former [e.g. chimpanzees], [33, 34]. This interpretation implies localised stimulus enhancement [*sensu* 35] in combination with an emulative, or goal directed, process [36] or observational conditioning [*sensu* 37]. Alternatively, there may be an exploratory phase early on, corresponding to an individual monkey's unsuccessful manipulations, prior to their option choices being biased by the successful behaviour of conspecifics.

Our option-bias method could potentially be widely applied within the field of social learning and culture. The approach circumvents the inherent problems arising from the lack of a controlled ‘demonstrator-observer’ scenario, tasks that afford few alternatives for solution, incomplete data, small group sizes and low statistical power. Thus the method may prove useful to other researchers attempting to distinguish social and asocial learning in social contexts and provides a new and potentially valuable tool for the identification of cultural traditions. The method could be deployed within controlled experimental and captive animal settings and, with the below caveats, to natural datasets too. However, it is important to emphasise the underlying assumption of the method – social learning leads to homogeneity of behaviour – and the consequent need for researchers to account for other factors (eg. genetics, ecology) responsible for homogeneity and to use a level of population analysis appropriate to the given context. For example, where a group is very large, or the mode of transmission is believed to be purely vertical, heterogeneity of behaviour may be expected between cliques, or genetically related sub-groups, necessitating analysis at this level. The method is applicable to instances where tasks have any number of options available for solution. Data on the presence or absence of a potential socially learned preference (i.e. one option, such as a diet choice) can be tested. The method could also be applied to natural behaviour for which there exist two or more variants. Naturally occurring phenomena that might fit this context include chimpanzee termite fishing where either one end or both ends of the tool (e.g. non-woody stem) are used and grooming traditions involving either the clasping of a branch or conspecific hand [38], [3, McGrew personal communication]. The approach is also suitable where there is only one motor pattern required to solve the task but variation in the ‘option’ choice within it [33]. The fact that the method does not require the researcher to record the inception and initial spread of the trait further enhances its utility in natural populations.

Importantly, the method can legitimately be applied in cases where there is an unequal prior probability of performing the two options, for instance, as a result of ecological or genetic variation; indeed, we find that the relative power of our method is enhanced in such cases compared to Fisher's exact test (see Supplementary material, S1.2). The method is not designed to distinguish unlearned from learned behaviour, and would have to be employed in conjunction with other approaches to partial out any influence of genetic variation on population-level differences in behaviour [e.g. 9]; the same holds for ecological variation. However the presence of genetic or ecological differences between populations in the probability of option use does not *a priori* rule out application of the method. Our analyses reveal that provided independent estimates of the probability of option use can be generated for each population, the method will generate reliable results. In cases where such estimates are not available, researchers are forced to apply the method on a smaller scale, at which such variation does not apply. We note here that the Monte Carlo simulation variant of the approach can be applied to detect social learning in a single population (see further explanation below). While the option-bias approach is not foolproof, and like any statistical approach is vulnerable to both Type I and Type II errors, our analyses reveal that it is associated with greater statistical power and lower error than alternative methods. Moreover, in comparison to the dominant ‘ethnographic method’, it allows the likelihood of social transmission to be evaluated according to consensual standards by computing precise estimates of the probability that chance or asocial processes could generate the observed patterns in the data.

The term ‘option-bias method’ refers to the use of observed homogeneity to infer social transmission, and not to the method deployed to bootstrap the asocial sampling distribution. While here we have placed emphasis on a variant of the method that utilizes randomization to compute asocial probabilities, we do not rule out the possibility that other methods for generating the asocial sampling distribution could prove useful in other circumstances. For instance, the Monte Carlo method described in the supplementary material (S1) has the added advantage that it can be used to detect a significant option bias in a single group of animals. Here, only one statistic (e.g *χ* ^2^) would be calculated from the observed data, but again the likelihood of any option bias detected being due to chance or asocial learning can be calculated by using simulation to bootstrap a probability distribution (note that any uncertainty in the parameters underlying asocial learning needs to be accounted for in the simulations, see S1 for more details). Moreover, within groups there are often sub-groups (e.g. matrilines) that can be incorporated into the analysis either as distinct groups in a population or used as a covariate to the single group analysis. Alternatively, data derived from individually tested animals could be resampled to this effect. Clearly, further work is needed to refine this method, and establish which variants will be most effective in which context. We are currently undertaking additional data collection to further validate the method and its various possible extensions.

In summary, the option-bias method provides researchers with a much-needed tool with which to assess the evidence for social learning in animal populations, as well as to investigate the learning strategies deployed, directly in their study animals. The procedure is potentially a significant technological development, which could prove of considerable value in assessing the validity of claims for culturally transmitted behaviour, particularly when used in conjunction with additional methods.

## Supporting Information

Table S1 Option-bias χ^2^ values for each trial used in the option bias analysis.
(0.29 MB DOC)

Material S1 Validation of the option-bias method through simulation.
(0.20 MB DOC)

Material S2 R functions for implementing an option-bias analysis.
(0.06 MB DOC)
