# Supplementary material for: Identifying Social Learning in Animal Populations: A New ‘Option-Bias’ Method
Source: PLoS One. 2009 Aug 6;4(8):e6541. doi: 10.1371/journal.pone.0006541 (PMC2717327; doi:10.1371/journal.pone.0006541)
Supplement: Table S1 — Option-bias χ2 values for each trial used in the option bias analysis. (0.29 MB DOC) [file pone.0006541.s001.doc]

**Table S1: Option bias χ^2^ values for each trial used in the option bias analysis.**

| **Trial*** | **Task** | **Group** | **Species** | **Group Size** | **χ^2^ value** |
| --- | --- | --- | --- | --- | --- |
| 1 | Round-box | 1 | *L. chrysomelas* | 3 | 2.27 |
| 2 | Round-box | 5 | *L. rosalia* | 3 | 2.19 |
| 3 | Round-box | 6 | *L. chrysomelas* | 5 | 1.65 |
| 4 | Round-box | 8 | *L. chrysomelas* | 4 | 0.24 |
| 5 | Round-box | 12 | *L. chrysopygus* | 7 | 0.048 |
| 6 | Round-box | 15 | *L. rosalia* | 3 | 0.091 |
| 7 | Round-box | 16 | *L. rosalia* | 7 | 0.143 |
| 8 | Round-box | 19 | *C. argentata* | 6 | 0.38 |
| 9 | Round-box | 23 | *C. geoffroyi* | 7 | 1.324 |
| 10 | Round-box | 25 | *C. geoffroyi* | 5 | 6.55 |
| 11 | Round-box | 26 | *C. argentata* | 8 | 2.91 |
| 12 | Flip-top | 1 | *L. chrysomelas* | 3 | 7.2 |
| 13 | Flip-top | 5 | *L. rosalia* | 3 | 2.78 |
| 14 | Flip-top | 6 | *L. chrysomelas* | 5 | 0.053 |
| 15 | Flip-top | 7 | *L. rosalia* | 6 | 20 |
| 16 | Flip-top | 8 | *L. chrysomelas* | 4 | 13.5 |
| 17 | Flip-top | 12 | *L. chrysopygus* | 7 | 1.38 |
| 18 | Flip-top | 14 | *L. chrysopygus* | 6 | 2.67 |
| 19 | Flip-top | 15 | *L. rosalia* | 3 | 11.3 |
| 20 | Flip-top | 16 | *L. rosalia* | 7 | 5.44 |
| 21 | Flip-top | 19 | *C. argentata* | 6 | 0 |
| 22 | Flip-top | 20 | *C. argentata* | 8 | 5.44 |
| 23 | Flip-top | 23 | *C. geoffroyi* | 7 | 3.6 |
| 24 | Flip-top | 25 | *C. geoffroyi* | 5 | 4 |
| 25 | Flip-top | 26 | *C. argentata* | 8 | 0 |
| 26 | Cylinder | 8 | *L. chrysomelas* | 4 | 16 |
| 27 | Cylinder | 12 | *L. chrysopygus* | 7 | 0.25 |
| 28 | Cylinder | 15 | *L. rosalia* | 3 | 6 |
| 29 | Cylinder | 16 | *L. rosalia* | 7 | 0.067 |

*Trial number distinguishes between trials and does not refer to the chronological order of task presentation – the table is sorted by task type.
