# Supplementary material for: Identifying Social Learning in Animal Populations: A New ‘Option-Bias’ Method
Source: PLoS One. 2009 Aug 6;4(8):e6541. doi: 10.1371/journal.pone.0006541 (PMC2717327; doi:10.1371/journal.pone.0006541)
Supplement: Material S1 — Validation of the option-bias method through simulation. (0.20 MB DOC) [file pone.0006541.s002.doc]

**S1: Validation of the option bias method through simulation**

We ran simulations of the asocial and social learning process for each task to validate the option bias method. These allowed us to assess the type 1 error rate and power of the option bias method, and compare its performance to other candidate statistical tests. The simulations use an established learning rule to compute how simulated individuals’ behaviour changed over time, following repeated exposure to the tasks.

# Each simulation had the same number of groups, containing the same number of individuals, as the monkey data described in the main text. The simulation assumed that, within a trial, each individual performed the same number of successful food extractions as that of the corresponding monkey for that task. However, the order in which particular individuals manipulate the task was randomised within each group. For a given manipulation, the probability of extracting food from each of two options, *A* and *B*, is determined by the following probabilities,

# $p(A)=V_{A}+\beta_{A}(1-V_{A}-V_{B})$, (1a)

# $p(B)=V_{B}+\beta_{B}(1-V_{A}-V_{B})$, (1b)

# where $\beta_{A}$and $\beta_{B}$are global parameters representing the underlying preference for each option and $\beta_{A}+\beta_{B}=1$ (unless stated otherwise we take $\beta_{A}=\beta_{B}=0.5$), whilst $V_{A}$and $V_{B}$ are variables representing the strength of association between the reward and each option (A and B), for the manipulating individual at the time of manipulation.$V_{A}$and $V_{B}$ are initially set to zero, but are altered through asocial and social learning.

# Asocial learning occurs after each extraction for the manipulating individual: there is a change in strength of association for the option used (option i) according to the Rescorla-Wagner learning rule,

# $\Delta V_{i}=\alpha(\lambda-V)$, (2)

# where $\lambda=1$, $0<\alpha<1$, $V=V_{A}+V_{B}$ and $V\leq\lambda$, and no change for the other option. $\lambda$ represents the maximum total association, and $\alpha$ is the rate at which learning occurs.

# Social learning occurs after each extraction for all non-manipulating individuals, according to a modified version of the Rescorla-Wagner learning rule,

# $\Delta V_{i}=\alpha s(\lambda-V)$, (3)

# where *s* is the strength of the social learning effect, relative to asocial learning, where $0\leq s\leq\frac{1}{\alpha}$. For example, when *s*=0, there is no social learning and when *s*=1, non-manipulating individuals learn as quickly through observation as the manipulating individual. In reality, it is improbable that all individuals will observe every manipulation, since their attention might not be on the manipulating individual at the time of extraction. We do not model this as a stochastic effect. Instead, the parameter *s* can be thought of as representing a combination of the probability that a given individual observes a manipulation and the effect that this observation has on the strength of its associations. Therefore, social learning in the model represents the expected (average) effect of another individual’s manipulation, taking into account the probability it is actually observed by the individual in question.

# *S1.1: Comparisons between methods*

# To compare candidate statistical techniques, we generated multiple data sets for each task, using the simulation procedure described above, and analysed them using the different methods. Plausible values of $\alpha$ were estimated for each task using data from the innovators’ (i.e. first solvers) first two option choices across groups. The estimate of $\alpha$ used the innovators’ first two extractions to minimise the effects of social learning – these individuals effectively had no one from whom to learn the solution of the task and so could reasonably be regarded as indicative of asocial learning performance. After the first extraction, the learning rate equates to the strength of association for the first option used, *V*_i_, (see eqn 2). Consequently, the probability of repeating the first option choice in the second extraction, $P_{same}$, is given by $\alpha+(1-\alpha)/2$, therefore

# $\alpha=2P_{same}-1$. (4)

# Thus $\alpha$ was estimated at 0.20 for the flip-top and cylinder tasks, and 0.54 for the round-box task. The type 1 error rate was estimated as the percentage of simulations for which the null hypothesis was rejected when *s*=0. Power was estimated as the percentage of simulations for which the null hypothesis was rejected when *s*>0, determined for different effect sizes (*s*=0.2, 0.4, 0.6, 0.8, 1), with 1000 simulations run for each value of *s*.

# The candidate statistical techniques considered were as follows:

# Randomisation technique. As described in the text, using a chi-square test statistic, but only 1000 randomisations were performed, to cut down computation time.

# GLM randomisation technique. A generalised linear model (GLM) with a binomial error structure and logit link function was fit to the data, with “group” as an independent variable using the R function glm (MASS package). The test statistic was the deviance explained by group. The null distribution was generated by randomisation in the same way as for technique1.

# LLM randomisation technique. A log-linear model (LLM) was used to analyse the contingency table instead of a chi-square statistic using the R function glm. The test statistic was the deviance explained by the group:option interaction. The null distribution was generated by randomisation in the same way as for 1. The results were always identical as for 2, but unlike 2 this approach can be extended to more than two options.

# GLMM. A generalised linear mixed model with “group” as a fixed factor and “individual” as a random effect was fitted to the data using the R function glmmPQL (MASS and nlme packages). Option bias was assessed using a likelihood ratio test (LRT) comparing the model with a null model not containing “group”.

# Fisher’s exact test. The data were reduced to a single binary datum for each individual, representing its preference for option *A* (1) or option *B* (0). Individuals with no preference were dropped from the analysis. This gave a new contingency table for *group x option*, which was analysed using Fisher’s exact test using the R function fisher.test.

# Monte Carlo simulation. We simulated the asocial learning process as described above (s=0) 1000 times to generate a null distribution for the chi-square statistic. We first assumed that $\alpha$ was known, and then re-ran the simulations estimating $\alpha$ from the simulated first-solver data as described above.

We also considered fitting a GLMM with nested random effects for “group” and “individual”. However simulations revealed that the group-level option bias becomes bimodal as *s* increases, whereas a random effects model assumes coefficients for levels of a random effect are drawn from a normal distribution. Consequently, we considered this approach inappropriate.

***S1.2: Effect of an underlying option preference***

To consider cases in which there is an underlying preference for one option over the other, we re-ran and analysed 1000 simulations using the randomisation technique (using the chi-squared test statistic) for the flip-top data with $\beta_{A}=0.75$ and $\beta_{B}=0.25$ for *s*=0 and *s*= 0.4. This was compared with the results with $\beta_{A}=\beta_{B}=0.5$ using the prop.test function in R. We found that the type 1 error rate (*s*=0) was unaffected ($\chi_{1}^{2}=0.22$, p=0.64) and that power was only very slightly reduced for *s*=0.4 (95% confidence interval: -7% to +0.5%). In contrast, for Fisher’s exact test power was significantly reduced for *s*=0.4 (95% confidence interval: -22% to -11%) though the type 1 error rate (s=0) was, again, unaffected ($\chi_{1}^{2}=0.32$, p=0.57).

***S1.3: Estimating Power***

We also used the simulations to estimate the power of the option bias method to detect specific effect sizes, defined by the value of the parameter *s*, for each task. However, the estimate of power depended on the assumed value of $\alpha$, and hence it was important to allow for our uncertainty in the value of this parameter. As described above,$\alpha$ can be estimated by calculating $P_{same}$ from the first-solver data. Therefore we can quantify our knowledge about $\alpha$ by using a prior to posterior Bayesian analysis for a binomial proportion, to generate a posterior distribution for $P_{same}$. We assume that $0<\alpha<1$, and consequently $0.5<P_{same}<1$, but have no other prior knowledge of $P_{same}$, so we used a uniform distribution, U(0.5,1) as the prior for $P_{same}$. A prior of U(0,1) generates a posterior of the form Beta(1+x, 1+*n*-*x*), where *n* is the number of trials in the data (the number of first solvers) and *x* is the number of observed successes (the number of times the first solver used the same option for its second manipulation) (Gelman *et al.,* 2004). Therefore a prior of (0.5,1) generates a posterior of the same form that is left-truncated at 0.5. We generated a distribution for $P_{same}$ for each task by randomly drawing values from a Beta(a,b) distribution, replacing any values <0.5 until we had 1000 values for each task. The values were transformed using equation 4 to give a distribution for $\alpha$. The binomial data used for each task were Flip-top: 6/10; Cylinder: 3/5; Roundbox: 10/13, giving Flip-top: a=7, b=5; Cylinder: a=4, b=3; Round-box: a=11, b=4.

For each value of $\alpha$ we ran the simulation and applied the randomisation technique (using the chi-squared test statistic) to the simulated data to generate a p-value. This yielded a joint distribution for $\alpha$ and p-values: the marginal distribution of p-values (reflecting our uncertainty in $\alpha$) is then just the distribution of p-values across all values of alpha. We calculated power as the proportion of p values <0.05. This procedure was repeated for each effect size (*s*= 0, 0.2, 0.4, 0.6, 0.8, 1).

**References**

Gelman, A., Carlin, J.B., Stern, H.S. & Rubin, D.B. 2004. *Bayesian Data Analysis (2^nd^ edition)*. Chapamn & Hall/CRC. Boca Raton, Florida.

R Development Core Team (2008). R: A language and environment for statistical computing. R Foundation for Statistical Computing, Vienna, Austria. ISBN 3-900051-07-0, http://www.R-project.org.
