# Supplementary material for: Identifying Social Learning in Animal Populations: A New ‘Option-Bias’ Method
Source: PLoS One. 2009 Aug 6;4(8):e6541. doi: 10.1371/journal.pone.0006541 (PMC2717327; doi:10.1371/journal.pone.0006541)
Supplement: Material S2 — R functions for implementing an option-bias analysis. (0.06 MB DOC) [file pone.0006541.s003.doc]

**S2: R functions for implementing an Option Bias analysis**

In the section S2.1 we provide functions to run an Option Bias analysis in the statistical language R (R Development Core Team, 2008). Here we provide details of each function, but we also refer the reader to section S2.2, where we provide an annotated example of an Option Bias analysis using these functions. Here we assume the data are for *n* individuals solving a task with *o* options:

**obData(group, options, idname, taskid)**

This function creates an object of class obData: this is the form the data needs to be in to run the Option Bias analysis. The arguments are:

**group**: a numeric vector of length *n*, giving the group membership of each individual in the data.

**options**: a matrix of dimension (*n* x *o*) giving the number of times each individual utilised each available option.

**idname**: an optional vector giving an identity for each individual.

**taskid**: a label for the task being solved (purely for future reference) defaulting to “1”.

**glmrand(data, simnumber=10000, plotHist=F, progress=F)**

Runs the randomisation test using the GLM randomisation technique described above.

**data**: an object of class obData.

**simnumber**: the number of randomisations to be used to generate the null distribution, defaulting to 10,000.

**plotHist**: if TRUE then the null distribution is plotted as a histogram with a vertical red line showing the observed value of the test statistic.

**progress**: if TRUE the number of each randomisation is displayed on the console, allowing the user to keep track of the progress of the randomisation process.

**llmrand(data, simnumber=10000, plotHist=F, progress=F)**

Runs the randomisation test using the LLM randomisation technique described above. Arguments are as for **glmrand**.

**chisqrand(data, simnumber=10000, plotHist=F, progress=F)**

Runs the randomisation test using the Chi-square test statistic, as used in the main text. Arguments are as for **llmrand**.

**simOB(data, alpha=1, s=0, bias= NULL)**

Simulates Option Bias data corresponding to a named obData object: the number of individuals, *n*, number of options, *o*, the number of manipulations by each individual and group membership match that of the specified obData object. The simulation is run as specified above.

**data**: an object of class obData.

**alpha**: a value for the parameter alpha

**s**: a value for the parameter s

**bias**: a vector of length *o* giving the underlying bias for each option. These can be input as a ratio of any arbitrary scale

**powerGivenAlpha(data, alpha, s, method="chisqrand", powersim=1000, simnumber=1000, significance=0.05, bias=NULL, plotHist=F, progress=F)**

Calculates the power to detect a specified effect size, *s*, for a specified set of data and a specified value of alpha. This is done by simulation as described above, through repeated calls to **simOB**.

**data**: an object of class obData.

**alpha**: a value for the parameter alpha

**s**: a value for the parameter s

**method**: the method of analysis used: “chisqrand”, “glmrand” or “llmrand”.

**powersim**: the number of simulations used to calculate power

**simnumber**: the number of randomisations used to generate the null distribution for each simulation.

**significance**: the significance level used to determine a significant result, defaulting to 0.05.

**bias**: as for **simOB**

**plotHist**: if TRUE a histogram of p-values is plotted.

**progress**: if TRUE the simulation number is printed to the console to allow the user to track progress.

**postPsame(fsolve, fsolvesame**)

Calculates a posterior distribution for alpha using a prior to posterior Bayesian analysis (see above). A vector containing the parameters of the Beta posterior distribution is returned.

**fsolve**: the number of first solvers

**fsolvesame**: the number of first solvers who used the same option for their second manipulation.

**sampleAlpha(fsolve, fsolveSame, n**)

Returns a sample from a Beta distribution calculated by calling **postPsame**.

**fsolve**: as for **postPsame**.

**fsolvesame**: as for **postPsame**.

**n**: the size of the required sample.

**powerSumOverAlpha(data, fsolve, fsolvesame, s, method="chisqrand", powersim=1000, simnumber=1000, bias=NULL, significance=0.05, output="power", plotDist=F, progress=F)**

Calculates the power to detect a specified effect size, *s*, for a specified set of data summing over uncertainty in alpha (see above). This is done by simulation as described above, through repeated calls to **simOB**. taking as **alpha** values generated using **sampleAlpha**.

All arguments are as for **powerGivenAlpha** or **sampleAlpha** except:

**output**: specifies what is returned: “power” returns a single value giving the percentage of simulations for which an effect was detected; “joint” returns a 2-column matrix containing the joint distribution of alpha and p-values; “marginal” returns a vector containing the marginal distribution of p-values.

**plotDist**: if TRUE works as for **powerGivenAlpha** unless **output= “joint”**, in which case a scatterplot giving the joint distribution of alpha and p-values is plotted.

**obFisher(obData)**

Applies a Fishers exact test to an object of class obData to test for an option bias as described above.

**obData**: an object of class obData

***S2.1: Option Bias Script (in R)***

setClass("obData",representation(group="vector",options="matrix",totals="vector",idname="vector",taskid="character"));

setMethod("initialize",

signature(.Object = "obData"),

function (.Object, group, options, idname=NULL,taskid="1",...)

{

#Create default names vector if none is provided, if it is, convert to a factor

if(is.null(idname)) idname<-(1:length(group));

totals<-apply(options,1,sum);

callNextMethod(.Object,idname=idname,group=group,options=options, totals=totals, taskid=taskid) }

)

#Function to create an option bias data object

obData<-function(group, options, idname=NULL,taskid="1"){new("obData",group=group,options=options,idname=idname,taskid=taskid)}

#Applies a randomisation test for option bias using a binomial regression. Can only be used for two-option tasks.

glmrand<-function(data,simnumber=10000,plotHist=F,progress=F){

if(dim(data@options)[2]>2) return("glmrand can only be used for two-option tasks. Use llmrand or chisqrand")

## Fit a glm to the data

rv<-data@options;

group<-data@group;

glmmodel<-glm(rv~as.factor(group),family=binomial);

OBstat<-anova(glmmodel)[2,2];

nullDist<-matrix(0,nrow=simnumber);

for(i in 1:simnumber){

newgroup<-sample(group,length(group));

glmmodel2<-glm(rv~(as.factor(newgroup)),family=binomial);

nullDist[i]<-anova(glmmodel2)[2,2];

if(progress==T){

cat(i,"\n");

flush.console();

}

}

if(plotHist==T){

hist(nullDist,breaks=20,xlab="OB Test Statistic",main="Null Distribution");

abline(v=OBstat,col=2);

}

pValue<-sum((na.omit(nullDist))>OBstat)/length(na.omit(nullDist));

return(c(OBstat,pValue));

}

#Applies a randomisation test for option bias using a log linear model

llmrand<-function(data,simnumber=10000,plotHist=F,progress=F){

## Fit a llm to the data

rv<-as.vector(data@options);

group<-data@group;

LLMgroup<-as.factor(rep(group,dim(data@options)[2]));

LLMoption<-as.factor(rep(1:dim(data@options)[2],each=dim(data@options)[1]));

llmmodel<-glm(rv~LLMgroup*LLMoption,family=poisson);

OBstat<-anova(llmmodel)[4,2];

nullDist<-matrix(0,nrow=simnumber);

for(i in 1:simnumber){

newgroup<-sample(group,length(group));

newLLMgroup<-as.factor(rep(newgroup,dim(options)[2]));

llmmodel2<-glm(rv~newLLMgroup*LLMoption,family=poisson);

nullDist[i]<-anova(llmmodel2)[4,2];

if(progress==T){

cat(i,"\n");

flush.console();

}

}

if(plotHist==T){

hist(nullDist,breaks=20,xlab="OB Test Statistic",main="Null Distribution");

abline(v=OBstat,col=2);

}

pValue<-sum((na.omit(nullDist))>OBstat)/length(na.omit(nullDist));

return(c(OBstat,pValue));

}

#Applies a randomisation test for option bias using a chi squared test statistic

chisqrand<-function(data,simnumber=10000,plotHist=F,progress=F){

group<-as.factor(data@group);

#Construct contingency table

contTable<-matrix(nrow=length(levels(group)),ncol=dim(data@options)[2])

for(i in 1:dim(data@options)[2]){

contTable[,i]<-tapply(data@options[,i],group,sum);

}

#Calculate expected

expected<-matrix(rep(apply(contTable,1,sum)/dim(contTable)[2],dim(contTable)[2]),nrow=dim(contTable)[1]);

#Calculate chi squared distances for each cell

chiDistances<-((contTable-expected)^2)/expected;

#Sum to get cho squared test stat

OBstat<-sum(chiDistances);

#Simulate null distribution

nullDist<-matrix(0,nrow=simnumber);

for(j in 1:simnumber){

newgroup<-sample(group,length(group));

contTable2<-matrix(nrow=length(levels(group)),ncol=dim(data@options)[2])

for(i in 1:dim(data@options)[2]){

contTable2[,i]<-tapply(data@options[,i],newgroup,sum);

}

#Calculate expected

expected2<-matrix(rep(apply(contTable2,1,sum)/dim(contTable2)[2],dim(contTable2)[2]),nrow=dim(contTable2)[1]);

#Calculate chi squared distances for each cell

chiDistances2<-((contTable2-expected2)^2)/expected2;

#Sum to get chi squared test stat

nullDist[j]<-sum(chiDistances2);

if(progress==T){

cat(j,"\n");

flush.console();

}

}

if(plotHist==T){

hist(nullDist,breaks=20,xlab="OB Test Statistic",main="Null Distribution");

abline(v=OBstat,col=2);

}

pValue<-sum((na.omit(nullDist))>OBstat)/length(na.omit(nullDist));

return(c(OBstat,pValue));

}

#Simulated option bias data from a specified model, input option bias object, alpha, s, underlying bias, return option bias object

simOB<- function(data,alpha=1,s=0,bias=NULL){

#Get number of options from data

noOptions<-dim(data@options)[2];

#Get group vector

group<-data@group

#Get number of groups

ngroups<-length(levels(as.factor(group)));

#Get number in each group

ingroup<-as.vector(table(group))

#Get total number of successes for each individual

successes<-data@totals

#Set no bias if none is provided. Standardise to sum to 1

if(is.null(bias)) bias<-rep(1,noOptions);

bias<-bias/sum(bias);

#index keeps track of which individual we are on throughout the group loop

index<-0;

## Cycles through groups

for (l in 1:ngroups){

trialorder<-vector();

## Defines and randomises the order in which individuals interact with the task

for (m in 1:ingroup[l]){

index=index+1;

trialorder<-c(trialorder,rep(m,successes[index]));

}

trialorder<-sample(trialorder,length(trialorder));

#Create matrix to record individual associations with each option

optassoc<-matrix(rep(0,ingroup[l]*noOptions),nrow=noOptions);

#Create matrix to record option chosen each manipulation

optcount<-matrix(rep(0,length(trialorder)*noOptions),nrow=noOptions);

## Simulates the learning process, and generates the chosen options accordingly

for (i in 1:length(trialorder)){

#Generate option from a multinomial distribution with probabilities determined by association strength

if(1-sum(optassoc[,trialorder[i]])>=0) option<-rmultinom(1,1,c(optassoc[,trialorder[i]],(1-sum(optassoc[,trialorder[i]]))*bias));

#Accounts for rounding errors when total association approaches 1

if(1-sum(optassoc[,trialorder[i]])<0) option<-rmultinom(1,1,c(optassoc[,trialorder[i]],rep(0,noOptions)));

#Records which option was chosen

for(j in 1:noOptions){

optcount[j,i]<-sum(option[c(j,j+noOptions)]);

}

#Updates association strengths by asocial (first term) and social (second term) learning

for(j in 1:noOptions){

optassoc[j,]<-optassoc[j,] +alpha*(1-apply(optassoc,2,sum))*optcount[j,i]*((1:ingroup[l])==trialorder[i]) +s*alpha*(1-apply(optassoc,2,sum))*optcount[j,i]*((1:ingroup[l])!=trialorder[i]);

}

}

temp<-matrix(nrow=noOptions,ncol=ingroup[l]);

for(j in 1:noOptions){

temp[j,]<-tapply(optcount[j,],trialorder,sum);

}

if(l==1){

opttable<-temp;

}else{

opttable<-cbind(opttable,temp);

}

}

obData(group=group,options=t(opttable),taskid=data@taskid);

}

#Functions to add

#Calculate power for a given value of alpha

powerGivenAlpha<-function(data,alpha,s,method="chisqrand",powersim=1000,simnumber=1000,significance=0.05,bias=NULL,plotHist=F,progress=F){

if(plotHist==T) record<-vector();

negative<-0;

positive<-0;

#Loop through the specified number of simulations for caluculating power

for (i in 1:powersim){

if(progress==T){

cat(i,"\n");

flush.console();

}

#Simulate data and run test

if(method=="chisqrand") pValue<-chisqrand(simOB(data,alpha=alpha,s=s,bias=bias),simnumber=simnumber)[2];

if(method=="glmrand") pValue<-glmrand(simOB(data,alpha=alpha,s=s,bias=bias),simnumber=simnumber)[2];

if(method=="llmrand") pValue<-llmrand(simOB(data,alpha=alpha,s=s,bias=bias),simnumber=simnumber)[2];

#Record P value if histogram is required

if(plotHist==T) record<-c(record, pValue);

#Count as significant or non significant

if(pValue>significance) {

negative<-negative+1;

}else{

positive<-positive+1

}

}

if(plotHist==T) hist(record,breaks=(1/significance),xlab="P value",main=NULL);

return(positive*100/(positive+negative));

}

#Calculate a posterior distribution for pSame, based on a uniform prior and the proportion of first solvers who choose the same option for their second task solution

postPSame<-function(fsolve,fsolvesame){

betaShapea<-1+fsolvesame;

betaShapeb<-1+fsolve-fsolvesame;

return (c(betaShapea,betaShapeb));

}

#Sample from a posterior distibution for alpha, calculated using postPSame and assuming a prior for pSame ~ U(0.5,1) i.e. everything below 0.5 is rejected

sampleAlpha<-function(fsolve,fsolvesame,n){

param<-postPSame(fsolve,fsolvesame);

pSameDist<-rbeta(n,param[1],param[2]);

while( sum(pSameDist<0.5)>0){

pSameDist<-(pSameDist>=0.5)*pSameDist+(pSameDist<0.5)*rbeta(n,param[1],param[2]);

}

alphaDist<-2*pSameDist-1;

return (alphaDist);

}

#Calculate power summing over uncertainty in alpha

powerSumOverAlpha<-function(data,fsolve,fsolvesame,s,method="chisqrand",powersim=1000,simnumber=1000,bias=NULL,significance=0.05,output="power",plotDist=F,progress=F){

#Generate a distribution for alpha

alphaDist<-sampleAlpha(fsolve,fsolvesame,n=powersim);

pValues<-vector();

negative<-0;

positive<-0;

#Loop through the specified number of simulations for caluculating power

for (i in 1:powersim){

if(progress==T){

cat(i,"\n");

flush.console();

}

alpha<-alphaDist[i];

#Simulate data and run test

if(method=="chisqrand") pValue<-chisqrand(simOB(data,alpha=alpha,s=s,bias=bias),simnumber=simnumber)[2];

if(method=="glmrand") pValue<-glmrand(simOB(data,alpha=alpha,s=s,bias=bias),simnumber=simnumber)[2];

if(method=="llmrand") pValue<-llmrand(simOB(data,alpha=alpha,s=s,bias=bias),simnumber=simnumber)[2];

#Record P value

pValues<-c(pValues, pValue);

#Count as significant or non significant

if(pValue>significance) {

negative<-negative+1;

}else{

positive<-positive+1

}

}

if(plotDist==T){

if(output=="joint") {

plot(alphaDist,pValues,xlab="Alpha",ylab="P value",main="Joint distribution");

abline(h=significance,col=2)

}else{

hist(pValues,breaks=(1/significance),xlab="P value",main=NULL);

}

}

if(output=="power") return(positive*100/(positive+negative));

if(output=="joint") return(cbind(alphaDist,pValues));

if(output=="marginal") return(pValues);

}

#Apply Fisher's test to an obdata object

obFisher<-function(obdata){

#Remove individuals who showed no clear preference

noPrefRemovedOptions<-obdata@options[(apply(1*(obdata@options==matrix(data=rep(apply(obdata@options,1,max),dim(obdata@options)[2]),ncol=dim(obdata@options)[2])),1,sum)==1),];

noPrefRemovedGroup<-obdata@group[(apply(1*(obdata@options==matrix(data=rep(apply(obdata@options,1,max),dim(obdata@options)[2]),ncol=dim(obdata@options)[2])),1,sum)==1)];

fisher.test(table(apply(noPrefRemovedOptions,1,which.max),noPrefRemovedGroup))

}

***S2.2: Option Bias Analysis Example***

#Example: cylinder data

#STEP 1: First create an option bias data object

#Input number of "blue" for each individual

blue<-c(0,0,0,3,0,2,2,0,0,0,1,0,4,0,2);

#Input total manipulations

totals<-c(6,3,7,4,3,6,2,1,5,1,1,1,4,2,7);

#Calculate "other" option

other<-totals-blue

#Check

other

#Combine as a matrix

options<-cbind(blue,other)

options

#Input group membership

group<-c(8,8,8,12,12,12,12,12,15,15,16,16,16,16,16)

#Now use obData function to create an obData object

cylinder<-obData(group=group,options=options,taskid="cylinder")

cylinder

#STEP 2: Use the randomisation methods to test for option bias

par(mfrow=c(3,1))

#Chi squared metric (1000 sims only to speed things up a bit). Outputs test stat and p value

chisqrand(data=cylinder,simnumber=1000,plotHist=T)

#glm metric

glmrand(data=cylinder,simnumber=1000,plotHist=T)

#llm metric

llmrand(data=cylinder,simnumber=1000,plotHist=T)

#STEP 3: Create a simulated set of data with the same group structure and manipulations as the cylinder dataset, specifying alpha and s. *Just for illustration*

simOB(data=cylinder,alpha=0.5,s=0.5)

#Now with an underlying bias 75:25 in favour of "blue"

simOB(data=cylinder,alpha=0.5,s=0.5,bias=c(75,25))

#STEP 3: Suppose we want to compare the power of our technique, assuming alpha=0.5 for different (global) underlying option biases. 100 "inner" simulations in the randomisation test itself, 100 "outer" to determine power (for speed's sake again)

#No bias

powerGivenAlpha(data=cylinder,alpha=0.5,s=0.5,method="chisqrand",powersim=100,simnumber=100,plotHist=T,progress=T)

#Strong bias

powerGivenAlpha(data=cylinder,alpha=0.5,s=0.5,bias=c(90,10),method="chisqrand",powersim=100,simnumber=100,plotHist=T,progress=T)

#STEP 4: Now suppose we want a more accurate estimate of power that takes into account our uncertainty in alpha

#First get an idea of the levels of uncertainty in alpha, using sampleAlpha, specifying the number of first solvers and the number that used the same option second time round

hist(sampleAlpha(fsolve=5,fsolvesame=3,n=100000))

#Get a power estimate that takes this into account

powerSumOverAlpha(data=cylinder,fsolve=5,fsolvesame=3,s=0.5,method="chisqrand",powersim=100,simnumber=100,plotDist=T,progress=T)

#Or we can get the joint distribution of alpha and p values by changing the output option from the default ("power") to "joint". This produces a scatterplot of the joint distribution and stores it in a matrix object "dist"

dist1<-powerSumOverAlpha(data=cylinder,fsolve=5,fsolvesame=3,s=0.5,method="chisqrand",powersim=100,simnumber=100,plotDist=T,progress=T,output="joint")

dist1
